# Hospital Expenditure.—The Commissariat

**Published:** 1896-07-25

**Authors:** 


### THE INSTITUTIONAL WORKSHOP.

### HOSPITAL EXPENDITURE.—THE COMMISSARIAT.

### XXI.—BEEF TEA.

In all hospitals beef tea constitutes a very heavy item of expenditure. Its cost shows a wide range of variation according to the mode of preparation and the price paid for the meat; and, small as the original variation in cost may be, the large amount required in a hospital makes even a fractional increase in the cost per pint important. In some hospitals ½ lb. to a pint, in others ¾ lb. to a pint, in others (the majority) 1 lb. to a pint, and in some for double strength as much as 2 lbs. to a pint are used. A hospital with a daily average of about 300 beds will use something like 30,000 pints of beef tea in a year, allowing for a third of the patients receiving a pint a day. The cost works out as follows:—

| Weight of Beef to 1 pint. | Peef per lb. | Cost per pint. | Total for 30,000 pints. |
|---|---|---|---|
| ½ lb. | 4d. | 2d. | £250 |
| ½ lb. | 6d. | 3d. | £375 |
| ¾ lb. | 4d. | 3d. | £375 |
| ¾ lb. | 6d. | 4½d. | £562 |
| 1 lb. | 4d. | 4d. | £500 |
| 1 lb. | 6d. | 6d. | £750 |
| *2 lbs. | 4d. | 8d. | £1,000 |
| 2 lbs. | 6d. | 1s. | £1,500 |

It will be seen then that it is worth taking a good deal of trouble to ascertain whether good tea can be made with American beef averaging 2d. per pound cheaper than home-killed beef, and also whether double strength beef tea at double cost really contains a double proportion of nutritive qualities.

Before preparing beef teas for the following analyses an attempt was made to ascertain whether any standard recipe for preparing beef tea existed among the London hospitals. It was conclusively proved that no such common standard is in use, and that in hardly two institutions is the mode of preparation and the proportion of meat used identical. The method adopted was to cut the meat in small pieces, add cold water in the proportions indicated below, and allow the meat to soak in it for an hour before applying heat. All fat and gristle were as far as possible, by Dr. Rideal's directions, omitted. It was then simmered gently, where the quantity prepared would admit, in closed jars in the oven, and, in experiments involving the preparation of very large quantities, in closed saucepans over a slow fire. In all cases the conditions were identical under which the respective specimens of English and American beef tea were brewed. In pouring off the beef tea as little straining as possible was used, and all the sedimentary matter was included in the liquid sent for analysis.

Samples A and B were made from 1 lb. Scotch and 1 lb. American beef respectively, soaked for an hour in one pint each of cold water, and then simmered for three hours in the oven in closed jars.† It may be added that after all the tea and sediment had been poured off the goodness of the beef was not by any means exhausted. The liquid did not set to a jelly when cold in either case. The colour after it had stood for a few hours was much the same in both samples, viz., an almost colourless liquid at the top, and a brownish sediment below; the sediment of the American tea was of a slightly darker tinge.

The report of Dr. Rideal on these two specimens is as follows:—

| | A. Scotch Meat. | B. American Meat. |
|---|---|---|
| | Grams per 100 c.c. | |
| Suspended matter | 0·977 | 0·988 |
| Total extract | 2·24 | 2·01 |
| Mineral matter | 0·46 | 0·47 |
| Total nitrogen | 0·224 | 0·212 |
| Albumen | 0·011 | 0·019 |
| Acidity in c.c. N/10 soda | 29·3 | 22·0 |

From the above analyses I am of the opinion that the two samples of beef tea do not show any appreciable difference, and it would be impossible to distinguish the origin of the two samples.

Owing to the fact that the infusion for three hours did not appear to have exhausted all the nutritive properties of the beef, the meat in the next experiment was simmered for twice the length of time, viz., for six hours. The proportion of meat was increased with a view to testing the food value of double strength beef tea, and a much larger quantity was prepared in order to render the chemical analysis less subject to chance. To eleven pounds of finely-minced Scotch (C) and American (D) beef respectively, six pints of water were added, and after cold infusion for an hour the whole was simmered for six hours continuously and then poured off; great care was taken to preserve the sediment, and every drop of liquid was carefully pressed out of the beef. When measured it was found that the quantity of liquid had materially increased by the extraction of the meat juices, the Scotch to 7 pints, and the American to 7½ pints. The difference in the ratio of increase may have been due either to the drier condition of frozen meat, which we have had occasion to notice in former experiments, or to some slight increase of evaporation from the American brew. Contrary to expectation, after standing all night, neither tea had set to a jelly.

REPORT.

The analyses of samples C and D are as follows:—

| | C. Scotch. | D. American. |
|---|---|---|
| | Grams per 100 c.c. | |
| Suspended matter | 2·975 | 2·348 |
| Total extract | 3·839 | 3·649 |
| Mineral matter | 0·632 | 0·659 |
| Total nitrogen | 0·529 | 0·450 |
| Albumen | 0·009 | 0·135 |
| Albumose | 1·447 | 1·238 |
| Nitrogen as flesh bases | 0·2189 | 0·205 |

Neither of these samples of beef tea was so acid as those first examined, and it will be noticed that so far as total extractives and mineral matter are concerned the English and American teas are practically identical in composition. The American tea was richer in albumen than the English tea, but the English tea was superior in containing a larger quantity of albumose and peptone.

The beef from which the above teas had been made remained, after all the liquid had been poured off, a dry mass of fibre, to all appearance entirely valueless. But in order to test whether any nutritive qualities still remained in it, four pints of hot water were added to each and left for several hours to cool. The result was that both these fresh samples, E and F, set to a

* The use of double strength beef tea is confined always to special cases.

† This recipe is after Bartholow, quoted by Dr. Burney Yeo.—"Food in Health and Disease."

solid jelly. These second brews were then simmered for a couple of hours, the liquid was strained off, and samples were handed to Dr. Rideal. Without subjecting them to the same exhaustive analysis as the other teas, he ascertained the proportion of nitrogen contained in each, and his report comparing the two brews will be read with great interest.

I also received two samples of beef tea made from the residue of the former teas, and called "second brews." These second-brew teas set to a jelly on cooling owing to the large percentage of gelatine they contain. I am of opinion that the setting of a tea is simply a measure of the amount of gelatine present in the fluid, and affords no criterion of its food value. These second brews contain possibly more gelatine than the original teas, but are likely to be deficient in the more easily soluble albumen and albumoses. The total nitrogen present in the second brews was higher than in the original teas.

| | Scotch. | American. |
|---|---|---|
| | Grams per 100 c.c. | |
| Total nitrogen first brew ... | 0·529 ... | 0·450 |
| Total nitrogen second brew ... | 0·630 ... | 0·584 |

The result goes to prove that a very large proportion of nutritious matter is unconsciously wasted in hospitals where it is considered unnecessary to make a second infusion of the meat. In many recipes for beef tea three hours is mentioned as the maximum period for the brew to simmer, but Dr. Rideal's analysis makes it certain that even after twice that time an important residue of nutritive substances is retained by the beef. It seems, in fact, more than likely that one infusion can never be sufficient to extract all the goodness out of the meat, since however carefully the liquid is expressed a good deal of nutritive matter is inevitably left sticking to the fibrous remains. To make a second infusion would, however, in many institutions be considered the very lowest depth of a cheeseparing policy, and the waste involved by this time-honoured tradition may be easily estimated by a comparison of the nitrogenous contents of first and second brews given above.

